# Pre- and post-home visit behaviors after using after-hours house call (AHHC) medical services: a questionnaire-based survey in Tokyo, Japan

**DOI:** 10.1186/s12873-021-00545-w

**Published:** 2021-12-15

**Authors:** Ryota Inokuchi, Kojiro Morita, Xueying Jin, Masatoshi Ishikawa, Nanako Tamiya

**Affiliations:** 1 Department of Health Services Research, Faculty of Medicine, University of Tsukuba, 1-1-1 Tenno-dai, Tsukuba, Ibaraki, 305-8575 Japan; 2 Health Services Research and Development Center, University of Tsukuba, 1-1-1 Tenno-dai, Tsukuba Ibaraki, 305-8575 Japan

**Keywords:** After-hours primary care, Quality, Triage, Illness severity, Out-of-hour medical services, Emergency department

## Abstract

**Background:**

After-hours house call (AHHC) medical services have been implemented in Japan to reduce ambulance use, as well as overcrowding at the emergency department (ED). Examining the pre-and post-home visit behaviors of those using AHHC medical services will provide insights into the usefulness of these services and help develop strategies to reduce ED visits and ambulance use further.

**Methods:**

This questionnaire-based study used data from anonymized medical records and internet-based questionnaires completed by patients who used AHHC medical services in Tokyo, Japan, between January 1 and December 31, 2019. The questionnaire comprised two questions: (1) What action would the patient have taken in the absence of AHHC services and (2) what action was taken within 3 days following the use of the AHHC services. In addition, following home consultations, AHHC doctors classified the patient’s illness severity as mild (treatable with over-the-counter medications), moderate (requires hospital or clinic visit), or severe (requires ambulance transportation).

**Results:**

Of the 15,787 patients who used AHHC medical services during the study period, 2128 completed the questionnaire (13.5% response rate). Individuals aged ≤15 years and 16–64 years were the most common users of AHHC services (≤15 years, 71.4%; 16–64 years, 26.8%). Before using the AHHC service, 46.4% of the total respondents reported that they would have visited an ED had AHHC services not been available (≤15 years, 47.8%; 16–64 years, 42.8%; ≥65 years, 43.6%). The proportion of patients originally planning to call an ambulance was higher among those in the older age groups (≤15 years, 1.1%; 16–64 years, 6.0%; ≥65 years, 20.5%). After using the AHHC services, most patients (68.1%) did not visit a hospital within 3 days; however, the proportion of patients who visited an ED and called an ambulance within 3 days increased with the severity of illness.

**Conclusions:**

Increasing AHHC medical services awareness among older adults and patients assessed as having severe illnesses regularly availing of AHHC services may help reduce ED visits and ambulance use.

**Supplementary Information:**

The online version contains supplementary material available at 10.1186/s12873-021-00545-w.

## Background

Non-urgent emergency department (ED) visits and ambulance calls are now global issues [1]. ED crowding not only prolongs timely intervention for critically ill patients but also increases the burden on hospital medical staff [2]. Recently, several countries have developed after-hours house call (AHHC) or out-of-hours medical services to solve the problem of excessive ED visits and ambulance use [3–8].

In Japan, several interventions, such as telephone consultation services and educational activities to promote appropriate ambulance use [9] have been introduced to reduce non-urgent ED visits and ambulance use. Moreover, charging patients for ambulance services has been recently considered [10]. Regardless, the number of ambulance calls and the duration of transportation have continued to increase [11]; thus, alternative methods to promote appropriate ED or ambulance use are being explored.

In 2016, a private AHHC service was established in Tokyo, Japan. This service provides consultations—via telephone triages— to patients assessed as needing hospital or clinical visits. Investigating the pre-and post-home visit behaviors of those who use AHHC medical services stratified by age and illness severity would elucidate the usefulness of these services and help develop strategies to reduce ED visits and ambulance use. However, previous studies have not examined the impact of AHHC service use by age and disease severity. Thus, we used patient data and patient-completed questionnaires to investigate these behaviors in patients using Tokyo’s AHHC medical service.

## Methods

This study used data obtained from anonymized medical records and internet-based questionnaires completed by patients who used AHHC medical services between January 1 and December 31, 2019. The study was reviewed and approved by the Research Ethics Committee of the University of Tsukuba (approval number 1527).

### Data sources

In this study, the anonymized clinical records of all patients who had used the AHHC services during the study period were reviewed, along with the questionnaires they completed. Information regarding patient gender, age, and illness severity were extracted from the medical records. The questionnaire consisted of two questions: what action the patient would have taken in the absence of AHHC services (stay home, wait for consultation until a hospital opens, visit ED, or call an ambulance) and what action was taken within 3 days following the use of the AHHC services (no hospital visit, visited an outpatient clinic, visited an ED, received another house call, or called an ambulance). For patients aged ≤15 years, a questionnaire was administered to parents or guardians who sought AHHC medical services.

### Health insurance system in Japan

Japan’s health insurance system provides universal coverage. It provides healthcare services, with the patient paying 10–30% of the medical fee, depending on the income and age of the insured [12]. In addition, many municipalities provide subsidies for patients below 15 years of age to cover their health care costs.

### Primary care system in Japan

Japan does not have an established general practitioner system similar to that in European countries [13]. Moreover, family medicine remains unpopular in Japan; thus, primary care is mainly provided by specialists, such as internists and pediatricians, in clinical settings after they have been trained in a hospital [14].

### Emergency care service system in Japan

Emergency hospitals in Japan are categorized as primary, secondary, or tertiary [15]. Generally, for primary care, holiday and night-time EDs in hospitals or clinics are available for non-severe conditions. Secondary hospitals provide emergency first aid for patients and, if necessary, inpatient care. Tertiary hospitals provide tertiary emergency medical and advanced critical care [16, 17]. Ambulatory patients have free access to any hospital facility, regardless of their symptoms. Meanwhile, when a patient calls an ambulance, patients are transported to a secondary or tertiary hospital depending on the illness severity, and 97.9% of callers are transported by ambulance to hospitals [https://www.fdma.go.jp/publication/hakusho/r1/items/r1_all.pdf]. Furthermore, ambulance expenses are covered by taxes [18, 19].

### AHHC medical Services in Japan

A private AHHC medical service (Fast Doctors, Shinjuku, Tokyo, Japan) has been operating in Tokyo since 2016. The company operates 7 days per week and provides out-of-hours services (i.e., 19:00–06:00 on weekdays, 18:00–06:00 on Saturdays, and 24 h per day on Sundays and holidays). Patients can access the services via a direct phone call or request an online consultation.

Following a telephone triage, instead of sending an ambulance, the service sends a doctor directly to the patient’s residence. The telephone triage involves a patient calling an emergency telephone consultation service and being classified into one of five categories (red, orange, yellow, green, or white) based on symptom acuity. The red category implies the presence of a life-threatening condition or one that is likely to worsen or change rapidly; orange reflects a condition requiring immediate hospital attendance as the symptom(s) may worsen over time; yellow requires a hospital visit as the symptom(s) may worsen over time; green does not have symptoms listed in the previous categories but requires a hospital visit; and white reflects symptoms that do not require a hospital visit [9].

The AHHC doctors conduct home visits for patients classified as orange and yellow; for those classified as red, an ambulance is called; patients classified as green are provided with information about nearby clinics or a primary hospital; and patients classified as white are provided appropriate advice for home observation.

After the consultation, the AHHC doctors classify the patient’s illness severity into one of the three following categories: mild (can be treated using over-the-counter medications), moderate (require a hospital or clinic visit), or severe (require ambulance transportation) [20, 21].

### Additional costs for an out-of-hours visit in Japan

In hospitals and clinics, if a patient visits out of hours, they incur an out-of-hours charge in addition to the regular medical fee. There are three types of out-of-hour charges (covered by Japan’s universal health care system), depending on when such services are sought: after hours (06:00–08:00 and 18:00–22:00 on weekdays; 06:00–08:00 and 12:00–22:00 on Saturdays), midnight (22:00–06:00), and Sundays or public holidays.

In an AHHC medical service, patients are charged the regular medical fee, an out-of-hour home visit fee (covered by Japan’s universal health care system), and transportation (0.27 USD per kilometer, up to 8.7 USD).

### Statistical analysis

We compared the patient characteristics (age, gender, and illness severity) between questionnaire responders and non-responders. As appropriate, Pearson’s chi-square test or Fisher’s exact test were used to compare categorical variables, and Student’s *t*-test or the Wilcoxon-Mann-Whitney test were used to compare continuous variables. Analyses were performed using JMP 14.3 statistical software (SAS Institute, Cary, NC, USA), and a value of *p* < 0.05 was considered statistically significant.

## Results

Of the 16,067 patients who received AHHC medical services during the study period, we excluded 280 owing to missing responses. Of the remaining 15,787 patients, 2128 returned completed questionnaires (13.5% response rate).

### Characteristics of responders and non-responders

In each group, patients aged < 65 were the most common users. Patients who completed the questionnaires were younger than the non-responders (6 years [IQR 2, 25] vs. 7 years [IQR 2, 30], *p* < 0.001) and had less severe symptoms than the non-responders (*p* = 0.012; Table 1). The gender ratio did not differ between age groups.

**Table 1:** Characteristics of responder and non-responder patients

	Responders (*N* = 2128)	Non-responders (*N* = 13,659)	*p*-value
Age (years), median [IQR]	6 [2, 25]	7 [2, 30]	< 0.001
Age group, N (%)			
≤ 15 years	1519 (71.4)	8237 (60.3)	< 0.001
16–64 years	570 (26.8)	5159 (37.8)	
≥ 65 years	39 (1.8)	263 (1.9)	
Gender			
Male (%)	1014 (47.7)	6578 (48.2)	0.67
Severity (%)			
Mild	1253 (58.9)	7627 (55.8)	0.012
Moderate	865 (40.7)	5926 (43.4)	
Severe	10 (0.5)	106 (0.8)	

*IQR* interquartile range

### Probable actions in the unavailability of AHHC service

Table 2 shows that the proportion of patients originally planning an ED visit was 46.4%. In each age group, most patients reported that they would have visited an ED if the AHHC services had not been available (≤15 years, 47.8%; 16–64 years, 42.8%; ≥65 years, 43.6%). Table 2 also indicates that, as the age group increased, the proportion of patients who would have called an ambulance also increased (≤15 years, 1.1%; 16–64 years, 6.0%; ≥65 years, 20.5%).

**Table 2:** Actions taken if the after-hours house call (AHHC) services had been unavailable

	Stayed home	Waited for a consultation until a hospital opened	Visited an emergency department	Called an ambulance
All patients, N (%)	208 (9.8)	875 (41.1)	987 (46.4)	58 (2.7)
Age group				
≤ 15 years (*N* = 1519)	132 (8.7)	645 (42.5)	726 (47.8)	16 (1.1)
16–64 years (*N* = 570)	73 (12.8)	219 (38.4)	244 (42.8)	34 (6.0)
≥ 65 years (*N* = 39)	3 (7.7)	11 (28.2)	17 (43.6)	8 (20.5)
Severity				
Mild (*N* = 1253)	143 (11.4)	543 (43.3)	541 (43.2)	26 (2.1)
Moderate (*N* = 865)	65 (7.5)	331 (38.3)	441 (51.0)	28 (3.2)
Severe (*N* = 10)	0 (0)	1 (10.0)	5 (50.0)	4 (40.0)

When stratified by illness severity, the proportion of those who had planned to call an ambulance increased with illness severity (mild, 2.1%; moderate, 3.2%; severe, 40.0%).

### Behavior of patients using medical services within three days of using the AHHC service

Regarding the use of medical services within 3 days of using the AHHC service, Table 3 shows that most respondents did not visit a hospital (68.3%), while the rest visited an outpatient clinic (27.0%) and an ED (2.8%), received daily home visits (1.0%), and called an ambulance (0.8%).

**Table 3:** Patient actions during the three days following the use of the after-hours house call (AHHC) service

	After using the AHHC service				
	Did not visit a hospital	Visited an outpatient clinic	Used a daily home-visit	Visited an emergency department	Called an ambulance
Expected response before using the AHHC service					
All (N = 2128)	1453 (68.3)	575 (27.0)	60 (2.8)	22 (1.0)	18 (0.8)
Remained at home (*N* = 208)	158 (76.0)	39 (18.8)	3 (1.4)	6 (2.9)	2 (1.0)
Waited for a consultation until a hospital opened (*N* = 875)	602 (68.8)	250 (28.6)	9 (1.0)	10 (1.1)	4 (0.5)
Visited an emergency department (*N* = 987)	662 (67.1)	270 (27.4)	7 (0.7)	41 (4.2)	7 (0.7)
Called an ambulance (*N* = 58)	31 (53.4)	16 (27.6)	3 (5.2)	3 (5.2)	5 (8.6)
Illness severity					
Mild (N = 1253)	853 (68.1)	355 (28.3)	10 (0.8)	26 (2.1)	9 (0.7)
Moderate (N = 865)	598 (69.1)	218 (25.2)	11 (1.3)	32 (3.7)	6 (0.7)
Severe (N = 10)	2 (20.0)	2 (20.0)	1 (10.0)	2 (20.0)	3 (30.0)

When stratified by their intended action before using the AHHC service, the proportion of patients who did not visit hospitals within 3 days of their AHHC visit decreased in the following order: staying at home (76.0%), waiting for a consultation (68.8%), visiting an ED (67.1%), or calling an ambulance (53.4%). Further, when stratified by illness severity, the proportions of patients who visited an ED and called an ambulance within 3 days increased with increasing illness severity.

## Discussion

The present study found that 1) the proportion of patients who had originally planned to visit an ED was > 40% in each age group, 2) the proportion of patients who had originally planned to call an ambulance increased with patient age, and 3) the proportion of those who visited an ED or called an ambulance within 3 days of using the AHHC service was low; however, the proportion of patients who visited an ED or called an ambulance within 3 days increased with illness severity.

Although several studies have reported analyses of patient factors or behaviors leading to the ambulance call, this is the first to describe patient behaviors before and after using the AHHC medical services stratified by age and illness severity.

### Probable patient actions in the absence of AHHC service

The present study showed that 46.4% of the respondents would have visited an ED if the AHHC service had not been available. This result reflects the proportions reported in a Dutch study (43%) [22] and in an Australian study (40.1%) [23]. Additionally, we found that most responding patients (68.1%) reported not visiting a hospital within the following 3 days after using the AHHC service; 2.8% visited an ED and 0.9% called an ambulance within that period. Similarly, an Australian study showed that, after an AHHC visit, 40% of patients did not require a follow-up and 8.4% of patients visited an ED [23]. Other studies have also reported declines in ED visits following the introduction of the AHHC services [24–27]. These findings indicate that AHHC services have reduced the number of ED visits and ambulance calls.

### Patient age and ambulance calling plans

We found that the proportion of those who had planned to call ambulances prior to contacting AHHC service personnel increased with age. Previous studies have shown that older adults are more likely to call an ambulance than younger persons [19, 28–30] and that older adults disproportionately rely on ambulances to transport them to an ED [31], supporting our results.

In the present study, patients aged < 65 years used the AHHC service the most. Many studies indicate different results regarding user characteristics. Some have reported that older patients mainly use AHHC medical services [4], and most users are aged < 65 years [32–34]. These findings suggest that the use of private AHHC medical services may depend on each country’s emergency medical care system and insurance service and any additional charges associated with using AHHC medical services. As stated above, in Japan, many municipalities provide subsidies for patients below 15 years of age to cover their medical costs; this circumstance might have made it easier for their parents to access the AHHC medical services.

We found the proportion of AHHC medical service users aged ≥65 years to be extremely low. This finding may demonstrate a delayed uptake because the patients are required to search the internet for the information necessary to contact the service. In Japan, a 2014 government white paper reported that the proportion of individuals aged 20–59 years who used the internet was > 91%. However, this proportion declined sharply among people aged > 60 years; the proportion of internet users who were > 75 years was < 50% [35]. Thus, as society ages and the number of older adults with internet access increases, the availability of AHHC services may curtail ED visits and ambulance use. Moreover, similar to other countries, if Japan’s AHHC services are integrated into the public emergency call services and the operators are able to request them, the ability of AHHC services to reduce ED visits and ambulance calls effectively may increase.

### Behavior within three days of using AHHC services

We found that the proportions of patients visiting an ED and calling an ambulance after using the AHHC medical service were high among those who had originally planned to visit an ED or call an ambulance. Moreover, this proportion increased with illness severity. Several studies targeting patients who called an ambulance have reported that the patients who felt high levels of urgency would call an ambulance even for minor illnesses [36]. Therefore, anxious patients may visit an ED or call an ambulance even after using an AHHC service. In contrast, we found that 40% of patients classified as having a severe illness would have elected to remain at home or waited until their hospital was open before seeking consultation if the AHHC medical service had not been available. This finding suggests that AHHC services may provide interventions before the illness becomes too severe. To test this hypothesis, future studies should survey patients with severe conditions who used an ambulance and investigate whether patients would use AHHC services if provided.

### Limitations

The current study had some limitations. First, our study involved a single AHHC medical service. However, this AHHC service involves more than 18,000 nightly visits annually and is the largest after-hours emergency service in Japan. Second, prior to dispatching an AHHC physician, all AHHC service providers offer telephone triage, which may cause a selection bias. Third, the response rate for the questionnaire was relatively low; thus, this study may have a potential sample selection bias. Finally, while the AHHC reduced the number of patients who had originally planned to visit an ED, patients who planned to stay at home and wait for a consultation until a hospital opened may have used the AHHC service because it was available. In addition, we did not compare total charges in the AHHC medical service and a hospital or clinic during out of hours. Therefore, cost-effectiveness analysis will be needed.

## Conclusion

The current study supports previously published works indicating the benefits of AHHC medical services for curtailing ED visits and ambulance use. Further, the study indicates that increasing AHHC medical services awareness among older adults and the use of AHHC services for revisiting patients with severe illnesses after an initial AHHC service visit may reduce ED visits and ambulance use. In addition, AHHCs may be useful for providing assistance before an illness becomes severe. Consequently, our findings may be helpful in facing the problem of ED overcrowding.

## Supplementary Information

**Additional file 1.**

## Data Availability

The datasets used and/or analyzed during the current study available from the corresponding author on reasonable request.

## Abbreviations

AHHC: After-hours house call
ED: Emergency department
IQR: Interquartile range
