# Supplementary material for: Pre- and post-home visit behaviors after using after-hours house call (AHHC) medical services: a questionnaire-based survey in Tokyo, Japan
Source: BMC Emerg Med. 2021 Dec 15;21:159. doi: 10.1186/s12873-021-00545-w (PMC8672620; doi:10.1186/s12873-021-00545-w)
Supplement: Supplementary file 1 — Additional file 1. [file 12873_2021_545_MOESM1_ESM.docx]

**Supplementary File: Patient Questionnaire**

Question 1.

Age______

Question 2.

Sex: Male, Female

Question 3.

What action would you have taken if the after-hours house call medical service had not been available?

1) Stayed at home

2) Waited for a consultation until a hospital opened

3) Visited an emergency department

4) Called an ambulance

Question 4.

What action was taken by you within three days following the use of the AHHC services?

1. No hospital visit
2. Visited an outpatient clinic
3. Visited an ED
4. Received another house call
5. Called an ambulance
